# Evaluation of Risk Perception of COVID-19 Disease: A Community-Based Participatory Study

**DOI:** 10.1017/dmp.2020.311

**Published:** 2020-09-02

**Authors:** Ezat Samadipour, Fatemeh Ghardashi, Nahid Aghaei

**Affiliations:** Non-Communicable Diseases Research Center, School Paramedical, Sabzevar University of Medical Sciences, Sabzevar, Iran; School of Nasibeh Nursing and Midwifery, Mazandaran University of Medical Sciences, Mazandaran, Iran

**Keywords:** community health planning, COVID-19, disease outbreaks, risk perception

## Abstract

**Objective:**

How people behave in a crisis depends on their understanding and evaluation of risk and vulnerability. Therefore, this study was conducted to investigate the Iranians’ risk perception of coronavirus disease (COVID-19).

**Methods:**

An online survey was applied, which resulted in the collection of information on demographics, the 5 dimensions of risk perception (cognitive, political, social, cultural, and emotional), and trust in the government among the Iranian users of social networks. Data were analyzed by descriptive and analytical tests of SPSS (IBM Corp, Armonk, NY) software, and confirmatory factor analysis of Amos software.

**Results:**

A total of 364 persons from 20 provinces completed the questionnaire during February 25 to March 2, 2020. More than 80% of the participants believed that negligence and lack of close supervision of the authorities have led to the spread of COVID-19. The mean (SD) risk perception was 58.77 (± 10.11), indicating the medium level of risk perception of people. The second-order confirmatory factor analysis also indicated that cultural dimension had the highest positive correlation (0.96), emotional dimension had the highest negative correlation (-0.65), and social dimension had the least correlation with the risk perception model (0.08).

**Conclusion:**

Iranians’ risk perception of the COVID-19 outbreak is not optimal, and it seems necessary to improve it.

The severe acute respiratory syndrome coronavirus 2 (SARS-CoV-2) is a new type of coronavirus and a biological natural hazard. This emerging hazard has begun from China (Wuhan) and overwhelmed the whole world in a few months. The control of the coronavirus disease (COVID-19) outbreak focused only on the identification, treatment, and isolation of the infected people, tracking and quarantine of their close contacts, reduction in the travels and avoidance of the semi-cooked meat, and promotion of the public participation to break the transmission chain.^1,2^

Since COVID-19 is a contagious disease and no treatment and vaccine have been found for the disease, it caused general precautions of high importance. Moreover, experiences with the control of the outbreak of communicable diseases, such as SARS, pandemic influenza, and swine flu epidemic, showed that the adopted strategies and results largely required the risk perceptions of people of those areas.^3,4^ However, according to the researchers’ recommendations, health and behavioral interventions need to change over time, depending on the behavioral in different groups.^5^

In the general literature, hazard appropriated behavior is considered as a disaster risk perception. It was also confirmed that negative consequences of disasters were related to low-risk perception.^6,7^ According to the literature, during disasters, decisions are on the basis of community risk perception of the policy-makers.^3,4^

The theory of rationality, bounded rationality, and protection motivation are the most relevant theories of risk perception. According to the rationality theory, people’s risk perception is based on the cost-benefit considerations.^8^ Moreover, the protection motivation theory considers new risks as unfamiliar and uncontrollable, which will motivate more protection and thus a higher perception.^9^ Most of disaster risk studies followed 3 popular theories of the “psychometric model,” “cultural theory,” and “social reinforcement framework.” According to the psychometric model, a key factor in people’s risk perception is “fear and unknown risk factor.” Cultural theory focuses on social organizations and social human activities. The theory of social reinforcement framework communicates the risks with psychological, social, institutional, and cultural processes.^10^ According to the natural disaster risk perception model of Iran, effective belief factors included will, authority, emotion, cognition, and political, social, and cultural effective factors. Preliminary research on the avian influenza risk perception has shown an inverse relationship between risk perception and performance beliefs.^11^ Efficacy beliefs in the early stages of a contagious and emerging disease may be a major communication challenge for crisis management managers. However, high-risk communication messages, which are not understood by the audience or contain risky conflicting messages, are ignored.^12^ Since risk management involves a multi-hazard, participatory approach, managers need to target programs according to people’s expectations. Social, economic, and cultural characteristics of the community, together with the ideology and worldview of its inhabitants, constitute people’s risk perception.^13^

Implementation of programs requires awareness and understanding of the stakeholders’ tendencies and concerns. Since controlling COVID-19 requires implementation of the disease control guidelines, including hygienic considerations (washing hands with soap and water or alcohol-based hand rub) and physical distance (as often as possible, keeping at least 1 meter [3 feet] from other people, and avoiding crowded places), by the maximum number of people (more than 80%), it is necessary to direct health care resources to maximize the impacts of the risk perception^14^ – although physical distance and the closure plan for schools, universities, and public centers have been implemented in most provinces of Iran in the early days of March. However, this study aims to examine the levels of risk perception of COVID-19 and to identify factors influencing it, during the early days of the official announcement of the prevalence of the disease in Iran.

## METHODS

### Participants and Procedure

This is a cross-sectional survey study, which was conducted during February 25, 2020, and March 2, 2020, the first week of the outbreak declaration of COVID-19 in Iran. The population of the study was social media users. According to the latest statistics, penetration of mobile Internet in Iran from September 2019 was 67 687 004 people, which include more than 76% of the population of Iran, half of whom are social network users.^15^

The convenience sampling method and snowballs were used to collect information. An anonymous online questionnaire in Persian was available at http://samadi.porseshnameonline.com/form/945 on social networks Telegram and WhatsApp. At this time, the Iranian Government recommended home confinement for the general population, therefore, a link of the online questionnaire was first disseminated to the Telegram and WhatsApp groups, in which the researchers were members, then the group manager was asked to pin the questionnaire link for a week, and they were encouraged to pass it. Sampling continued in the form of snowballs, in which each participant helped to publish the questionnaire link by placing the questionnaire link in other social groups of which he was a member, such as the social group of family or friends. Thus, the questionnaire was simultaneously completed in different groups across the country. That way, a snowball sampling strategy, focused on recruiting the general public living in the whole country during the epidemic of COVID-19, was used.

Prior to the completion of the questionnaire, informed consent was obtained from all participants included in the study. Notably, the users participated voluntarily in the project, and they could be excluded from the study by selecting the “Complete” option at each stage of the questionnaire completion. All ethical items have been observed in this study in accordance with the ethical principles in human research of the Declaration of Helsinki, and have been approved by the Ethics Committee of Sabzevar University of Medical Sciences with the ethical code: IR.MEDSAB.REC.1398.119.

### Inclusion/Exclusion Criteria

The inclusion criteria were being Iranian, users of social media, and the willingness to participate in the study. The exclusion criteria were the unwillingness to complete the questionnaire.

### Questionnaire Design, Validity, and Reliability

The research instrument was a 3-part questionnaire with 34 items. The first section included 5 items about participants’ demographic information, including age, gender, degree, occupation, and place of residence. The second section included 3 items about participants’ trust in the government, including previous warnings from authorities, negligence, and the lack of supervision.

The 3 sections, including 26 items to evaluate the perception of risk, were prepared based on Samadipour’s risk perception model.^13^ Content validity was confirmed by 5 professors in health in disasters and emergencies. The risk perception questionnaire included 5 dimensions: cognitive (5 questions), political (5 questions), social (4 questions), cultural (6 questions), and emotional (6 questions).

The responses are rated on a 5-point in Likert scale ranging from 1 (”completely disagree”) to 5 (”completely agree”). The minimum and maximum scores of the questionnaire was 26 and 130, respectively. The total risk perception score was used to perform the inferential statistics. Interpretation and scoring of total risk perception were based on low (26–51), moderate (52–77), good (78–103), and very good risk perception (> 103).

Cronbach’s alpha coefficient of 0.787 confirmed the questionnaire reliability. In addition, construct validity was confirmed by the confirmatory factor analysis (CFA).

### Statistical Analysis

Descriptive statistics was used for categorical variables, such as demographic and risk perception. Analytical statistics, Spearman’s, was used to measure the correlation between demographic variables and the risk perception score. The significance level was set at 0.05. Analyses were performed in SPSS Statistics, Version 16.0 (IBM Corp, Armonk, NY).

CFA was used to calculate the validity questionnaire and the correlation of risk perception dimensions. The goodness of fit for each model was assessed using the Satorra–Bentler scaled chi-square, the Incremental Fit Index (IFI), and the Comparative Fit Index (CFI). A non-significant chi-square and values greater than 0.90 for the GFI, IFI, and CFI are considered to reflect an acceptable model fit (IBM SPSS, Amos version 23).

## RESULTS

The questionnaire was viewed 1265 times and filled by 364 persons. The results of the Kaiser Meyer Olkin (0.834) and the Bartlett’s test (0.000) confirmed the adequacy of the sample for the CFA.

**FIGURE 1:**
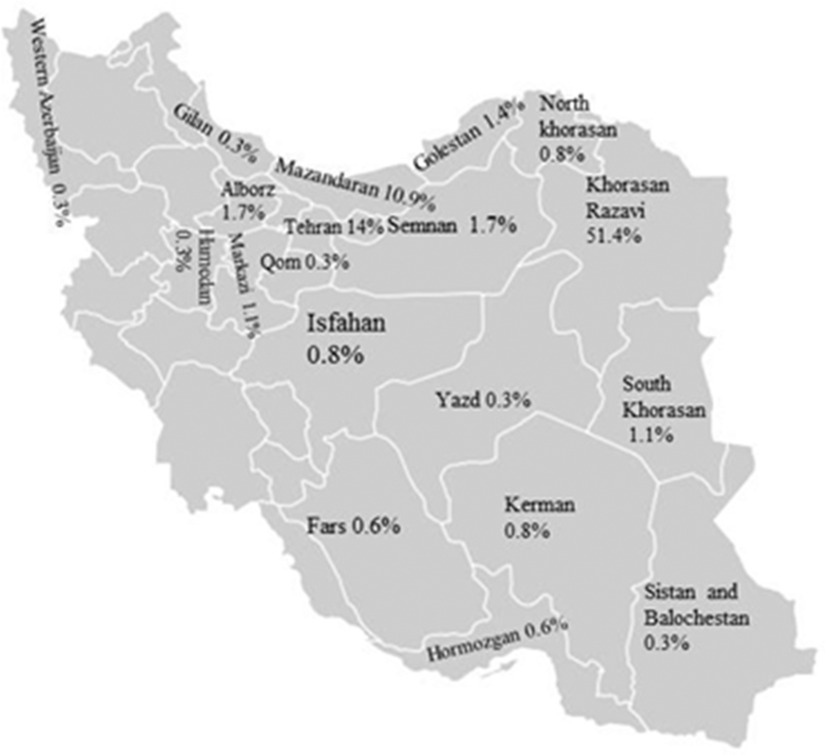
Participants’ Provinces of Residence.

Participants of the study were the social media’s users from 20 provinces of the country, most of whom were from Khorasan Razavi (51%), Tehran (14%), and Mazandaran (10.9%) Provinces (Figure 1). A majority of the participants were female (55.3%) with the age range of 20–39 years old (67.5%), and 36% were students (Table 1). The mean (SD) risk perception was 58.77 (± 10.11).

**TABLE 1:** The Demographics of Participants

Variable		N (%)
Gender	Male	154 (42.2)
	Female	201 (55.3)
	Missing	9 (2.5)
Age	10-19	21 (5.8)
	20-29	162 (44.5)
	30-39	91 (25.0)
	40-49	50 (13.7)
	50-49	22 (6.0)
	60 ⩾	4 (1.1)
	Missing	14 (3.8)
Job	Student	131 (35.9)
	Teacher	48 (13.2)
	Employee	87 (23.9)
	Unemployed	16 (4.4)
	Private job	22 (6.0)
	Healthcare	34 (2.7)
	Retired	10 (2.7)
	Missing	16 (4.4)
	Total sum: 364	

### Demographic Characteristics and Risk Perception

According to Spearman’s correlation test, no significant relationship was found between demographic variables and risk perception; however, only a weak correlation (R = 0.198) was observed between age groups and risk perception score (*P* = 0.000).

### People’s Trust in the Government

The other finding of this study is that, among total participants (358), only 106 (29.6%) agreed that the previous hazard warnings of the authorities were true and more than 80% of them agreed that the authorities’ negligence and lack of supervision influenced the initial prevalence of COVID-19 (Table 2).

**TABLE 2:**
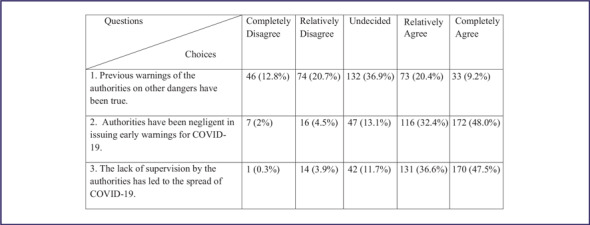
People’s Trust in the Government

### Dimensions of Risk Perception

**FIGURE 2:**
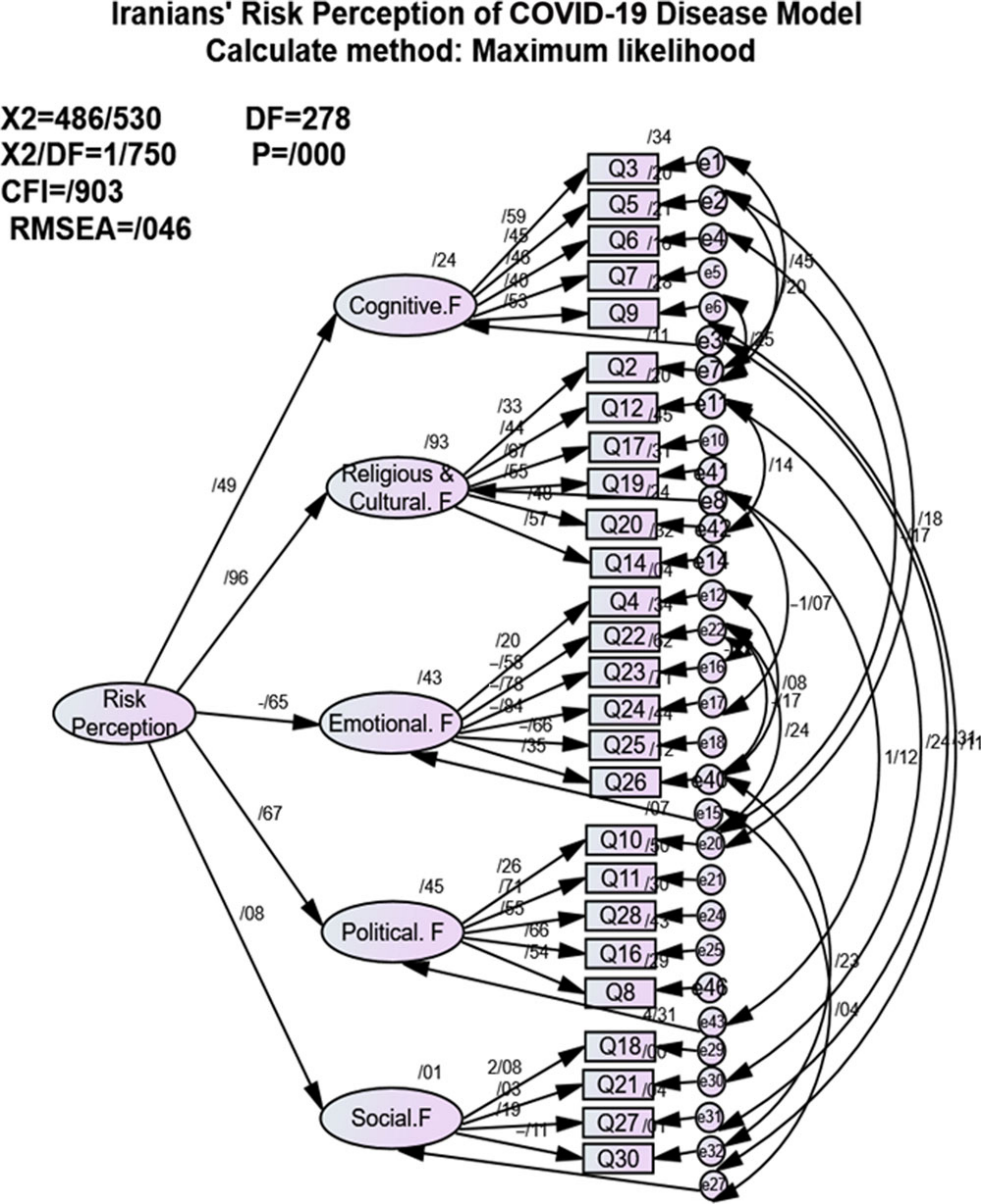
Iranians’ Risk Perception of COVID-19 Model.

CFA was used to confirm the validity of each item of the questionnaire. Results obtained from the first-order factor analysis confirmed the validity of 5 constructs of cognitive, emotional, social, political, and religious and cultural factors (Figure 2). Table 2 lists the measured structural indices and acceptable values of the indices. According to Hu and Bentler (1999), the guidelines followed for the valuation of the fit indices of the models tested were NFI, CGI, and TLI with a 0.95 cutoff point, SRMR with a 0.08 cutoff point, and RMSEA with a cutoff point of 0.06 (Table 3).^16^

**TABLE 3:**
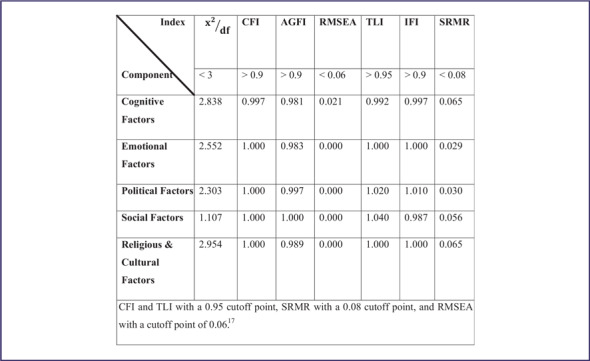
CFA Results for Dimensions of the Risk Perception (Measured Indexes)

Moreover, results of the second-order factor analysis showed that, according to the graphical model, cultural and religious factors had the highest positive correlation (0.96), emotional factors had the highest negative correlation (-0.65), and social factors had the least correlation with the risk perception model (0.08) (Figure 2).

## DISCUSSION

Risk perception is a dynamic concept and one of the most difficult and ambiguous categories of social vulnerability, which needs special attention to control COVID-19. Since the public participation, more than ever, is the only known way to inhibit the epidemic, decisions made at the time of disaster always were driven by the perceived risk of the affected population and policy-makers.^14,15^ In addition, it was found that people’s evaluation of risk depends on a number of factors, including direct and indirect experience and psychological mechanisms for judging risk.^17^

### People’s Trust in the Government

More than 80% of the participants believed that negligence and lack of close supervision of the authorities have led to the spread of COVID-19 disease. It is a sign of people’s distrust of the officials’ actions. Lack of public confidence in the warnings of officials is evident in the current behavior of the community, such as the lack of implementation of prevention guidelines and the lack of physical distance. The authorities’ lack of confidence in people’s understanding and ability to hear the facts and their non-participation in the preventive actions may be one of the reasons for the disease spread.

It should be noted that both people and authorities have a key role in disaster risk reduction, but different approaches to risk perception cause a gap between the goals and outcomes. Therefore, implementation recommendations of the disaster risk reduction programs should not be disregarded. On the other hand, lack of trust in the authorities’ warnings leads to failure in the application of the guidelines and reduction of physical distance. It was also found that trust in managers is one of the essential components of the community risk perception so that a direct relationship was reported between risk perception and trust in urban crisis management.^18-20^ Consequently, community-based disaster management improves trust, increases risk perception, and follows the epidemic control recommendations. Hence, it seems necessary for the authorities to create a mutual trust by revealing the facts to people.

### Risk Perception and Factors Affecting Risk Perception

Risk perception correlated significantly with the adoption of preventive health behaviors in all countries.^21^ Therefore, identifying the factors that affect it and trying to increase the perception of risk can help control COVID-19.^22,23^

The results of the study showed that the score of people’s risk perception of COVID-19 is moderate; also, cultural, emotional, social, cognitive, and political factors influenced the risk perception of the COVID-19 epidemic hazard.

### Religion and Culture

With regard to the findings, religious and cultural factors had the highest positive correlation (0.96) with the Iranians’ risk perception of COVID-19. This result was consistent with a study conducted by Chester on the impact of religion and religious beliefs on the risk perception and disaster risk management.^24^ On the one hand, Islam is very theologically varied, and many commentators have emphasized that Islam’s view of suffering is entirely to return people to their religious teachings.^25,26^ On the other hand, since most Iranians are Muslims, people’s responses to disasters depend on their religion and culture. Therefore, the role played by the clergy and cultural authorities of the country is of high importance in the risk perception of COVID-19. Hence, it is necessary to pay special attention to this role in order to control COVID-19 as soon as possible with a proper social function.

### Emotion

Emotional factors with a relatively strong inverse correlation indicated the destructive role of emotions in the perception of risk ahead. It is notable that studies of risk perception based on the psychological patterns directly considered the role of emotions in risk perception. For example, Slavic (2004) argued that risk analysis without understanding emotions such as anger, humiliation, fear, satisfaction, guilt, embarrassment, worry, pessimism, and optimism could not lead to risk perception.^22-26^ Therefore, although it seems necessary to create real and effective feelings about risk perception, their extent is not clear and thus further studies should be performed in the field.

### Social Factors

According to the analyses, the weakest correlation was related to social factors, which may be due to the type of hazards and prevention guidelines, which required the reduction of the social interactions and observation of the physical distance. Moreover, society has a great influence on the people’s lives and risk acceptance in the Iranian community, and this influence is increasingly evident with the expansion of urbanization and communication.

Although it is fair to say that many social groups were specifically created in the last few weeks to educate prevention protocols and induce hope and a proper perception of the risk of COVID-19, it seems that these actions, both quantitatively and qualitatively, did not respond to the social arousal of risk perception and thus further studies should be conducted in this regard.

### Political Factors

As demonstrated by the studies, political factors with a correlation of 0.67 could have an effective role in the perception of the Iranians’ risk of COVID-19. Studies have shown that people, who have a higher trust in the government, lose their risk tolerance if discredited and thus risks may be insignificant to them. Therefore, improving the government’s credibility is the most important factor in the risk tolerance of a community and thus it is recommended to reduce the high-risk behaviors in people, especially at low economic levels, strengthen local government credibility, and increase risk communication.^6,19,27-30^ According to a community-based educational need assessment study, responding to the health and well-being needs of people should be a priority for public institutions.^31^ Hence, it is imperative that government officials use all available means to increase their perceptions of risk and self-care capacity, so that they can help control the disease as quickly as possible.

### Cognitive Factors

Analyses indicated that cognitive factors had a moderate correlation with the risk perception. In fact, unawareness of the disease hazards may be due to different types of hazards and emergence of COVID-19. Although many studies have identified cognition as effective in risk perception, studies with more emphasis on floods, famines, and earthquakes have found little or even negative association with the risk perception.^17,29,32^

### Limitations

Due to the specific conditions of the disease epidemic, the study was conducted online. Therefore, the social media’s users voluntarily participate in the study, and they were ensured of the confidentiality of the data. Since the questionnaire was first placed in groups of friends, acquaintances, and colleagues of researchers, the questionnaire was distributed through these users. As a result, it was possible that various population groups would be missed.

## CONCLUSION

According to the results of this study, people had a moderate risk perception of COVID-19. Five factors – cultural, political, emotional, cognitive, and social – contributed to the Iranians’ risk perception of COVID-19. Therefore, it is suggested that authorities try to increase the Iranians’ risk perception by strengthening the trust between themselves and people and using modern technology and social medias’ facilities, and try to control the disease with proper orientation to these factors.

It is hoped that the results will be used to increase public risk perception of COVID-19 and thus help control the disease.
